# The impact of an interventional counselling procedure in families with a BRCA1/2 gene mutation: efficacy and safety

**DOI:** 10.1007/s10689-015-9854-4

**Published:** 2016-01-09

**Authors:** Erica Sermijn, Liesbeth Delesie, Ellen Deschepper, Ingrid Pauwels, Maryse Bonduelle, Erik Teugels, Jacques De Grève

**Affiliations:** Familial Cancer Clinic and Medical Oncology, University Hospital Brussels, Laarbeeklaan 101, 1090 Brussels, Belgium; Research Unit of Department of Internal Medicine, University Hospital Ghent, De Pintelaan 185, 9000 Ghent, Belgium; Department of Public Health, Biostatistics Unit, Ghent University, De Pintelaan 185, 9000 Ghent, Belgium; Department of Medical Genetics, University Hospital Brussels, Laarbeeklaan 101, 1090 Brussels, Belgium

**Keywords:** Predictive genetic counselling, BRCA gene mutation, Procedure, Efficacy, Safety

## Abstract

*Background:* Predictive genetic testing has high impact on cancer prevention for *BRCA* carriers and passing this information in *BRCA* families is important. Mostly, this is proband-mediated but this path is defective and denies relatives lifesaving information. *Objective:* To assess the efficacy/safety of an intervention, in which relatives are actively informed. *Design:* Sequential prospective study in new *BRCA* families. The proband informed relatives about predictive testing (phase I). After 6 months, a letter was sent to adult relatives who had not been reached (phase II). Then a phone call was made to obtain a final notion of their wishes. All subjects received psychometric testing (State-Trait Anxiety Inventory, STAI), an interview and routine counselling. *Results:* Twenty families were included. Twenty-four of the relatives could not be reached, 59 were ‘decliners’, 47 participated by the proband and 42 by the letter. Predictive testing was performed in 98 % of the participants of which 30 were mutation carriers. The intervention is psychologically safe: the 95 % CI for the estimated mean difference in STAI DY1 between phase II/I subjects (mean difference −1.07, 95 % CI −4.4 to 2.35, *p* = 0.53) shows that the mean STAI DY1 score (measured at first consult) for phase II is no more than 2.35 units higher than for phase I, which is not relevant. *Conclusions:* A protocol directly informing relatives nearly doubles the number of relatives tested and is psychologically safe. This should lead to a change in counselling guidelines in families with a strong germline predisposition for cancer.

**Electronic supplementary material:**

The online version of this article (doi:10.1007/s10689-015-9854-4) contains supplementary material, which is available to authorized users.

## Introduction

Breast cancer is the most common cancer affecting women. A familial predisposition exists in 5–10 % of all breast cancer cases, of which 15–20 % is due to germline mutations in the *BRCA1/2* genes [1]. Women carrying a *BRCA1* mutation have a cumulative risk of 57–65 % of developing breast cancer by 70 years, and those with a *BRCA2* mutation have a risk of 45–57 % [2–4]. Female *BRCA* carriers also have an increased risk of developing ovarian cancer, with a cumulative risk by 70 years of 39–59 % for *BRCA1*, and of 11–18 % for *BRCA2* carriers respectively [2–4]. Male *BRCA1/2* mutation carriers also have an increased, albeit significantly lower risk of breast and prostate cancer [5]. Both men and women with *BRCA1/2* mutations also have increased risk for other cancers, such as pancreatic cancer.

Predictive genetic testing is an important tool for advising *BRCA1/2* mutation carriers on preventive management strategies. It is therefore crucial that possible mutation carriers have maximal access to the option of such predictive testing. In a ‘non-directive’ counselling approach, widely used by most genetic counsellors throughout the world, the dissemination of information on a predictive genetic test within affected families is mainly via the proband as the unique interlocutor. With that strategy the transfer of information from probands to their relatives is highly defective [6–8]. This contrasts with the high interest in becoming informed about the genetic risk and the availability of predictive testing by the large majority of relatives which were not previously informed [7]. The availability of life-saving prevention strategies for mutation carriers, of specific treatments such as PARP1 inhibitors, and of pre-implantation genetic diagnosis (PGD) makes ‘the right to know’ a very prominent issue [9].

The aim of this study was to evaluate the efficacy, feasibility and safety of a stepwise interventional approach which aims to actively inform relatives at risk in families newly diagnosed with a *BRCA1/2* gene mutation.

## Methods

This study is a prospective single center study in newly diagnosed *BRCA1/2* mutant families. The study was performed in the Familial Cancer Clinic of University Hospital Brussels (UZ Brussel), Belgium, and was approved by the Ethics Committee of the University Hospital Brussels.

### Families/subjects and procedures

In the period from 2006 until 2012, families with a newly diagnosed *BRCA* mutation, were recruited for the study. Possible mutation carriers, as identified from the family tree, were included. Exclusion criteria were: younger than 18 years, possible psychiatric illness or other serious active illness. This study was an extension of the conventional counselling procedure. Probands first receive information about the various aspects of hereditary breast/ovarian cancer (HBOC), the possibility for a predictive mutation search, the consequences of finding a mutation in terms of cancer risks, and the preventive measures that can be taken, tailored to the subjects’ profile. Subjects in a reproductive age were also informed about options for PGD. After the identification of a *BRCA1/2* mutation, preventive measures were further discussed.

**Fig. 1:**
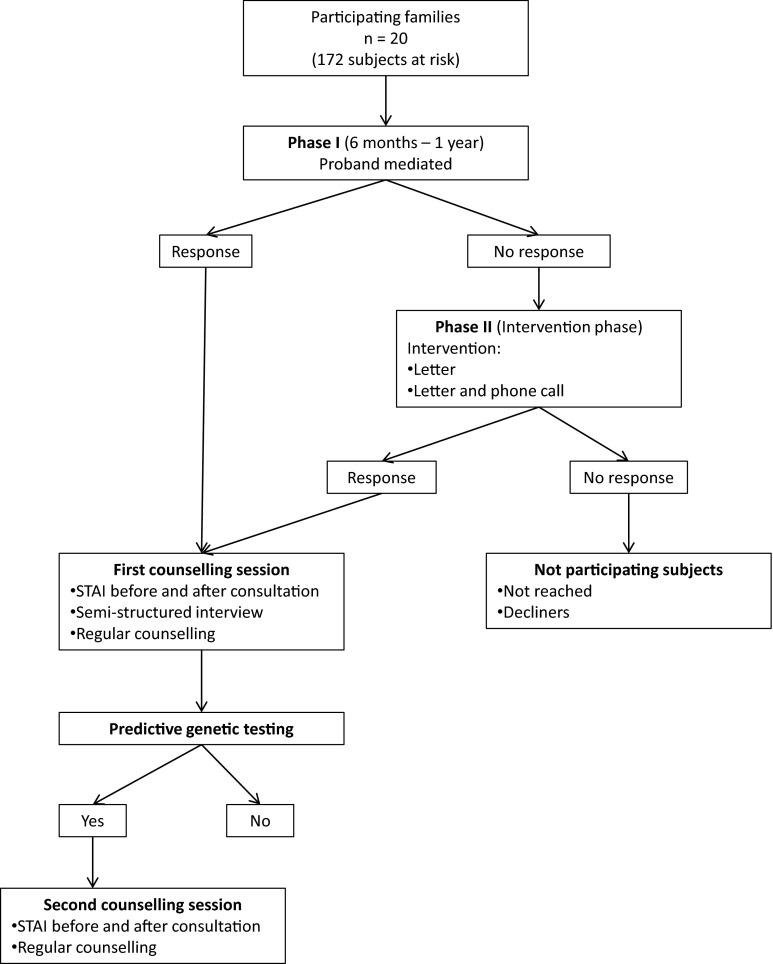
Study design

A two-phased protocol was then initiated in this study (Fig. 1). The study was explained to the proband and written informed consent was provided.

In the first phase of the study, the standard procedure was followed. There was no direct intervention of the counselling team towards other relatives. The proband was asked to inform other at risk relatives and to advise them to seek information at the Familial Cancer Clinic. The counsellor discussed in detail the familial pedigree, for deciding which at-risk relatives should be informed (all first, second, and more than second degree relatives which could be possible mutation carriers and without having exclusion criteria). The proband was provided with contact details of the Familial Cancer Clinic to distribute to the at risk relatives. Possible barriers in communicating this information, which can be mentally challenging, were discussed with the proband in advance. The proband was explained that she/he should be prepared to possible negative interactions. It was also emphasized that the proband should not feel obliged to inform relatives, and should only inform relatives if feeling comfortable in doing this. The proband also had the possibility to contact the Familial Cancer Clinic to discuss difficult situations. The proband had a second visit after 6 months to discuss the status of informing relatives and the difficulties which were encountered. Next researchers asked for any useful contact details of relatives not yet successfully contacted. Contact details were also validated or obtained from other sources such as a public Belgian address/phone number registry. In the second phase an informative letter was sent to at-risk relatives, who had not yet come forward in first phase, or could not be contacted by the proband. The letter informed about the familial cancer risk, the availability of a predictive genetic test and the option of having subsequent counselling. In all of this, the anonymity of the proband was preserved. Contact details as well as a reply questionnaire, probing the acceptability of the approach were provided. Attitudes of the family members to direct contact were assessed with a reply questionnaire which was sent together with the informative letter. If there was no further reaction within another 6 months, researchers tried to contact these relatives by phone to have a final ascertainment of their wishes.

All family members which came forward to the Familial Cancer Clinic (first or second phase) followed the same protocol. In this protocol the State-Trait Anxiety Inventory for adults by Charles D. Spielberger (STAI) [10, 11] was administered. The STAI is a widely used questionnaire and validated in Dutch [12]. It was developed to measure anxiety referring to a transitory emotional state, prompted by external or internal stimuli (state anxiety) (STAI DY1) or anxiety corresponding to a stable personality disposition (trait anxiety) (STAI DY2) [10, 11]. Each scale consists of 20 statements scored on a 4-point Likert scale. Response categories range from 1 (not at all) to 4 (very much so). After summing up the scores for the single items, the total scores on each scale ranges between 20 and 80. Higher scores are an indication for greater anxiety levels.

At the beginning of the first counselling session the STAI DY1 and STAI DY2 was performed. Within the group of family members which came forward to the Familial Cancer Clinic by second phase, we also asked actively at first contact how they experienced the direct contact by letter/phone call. Then the counsellor performed an in-depth semi-structured interview, in which comprehensive questions were discussed about the various aspects of HBOC, to end with a regular counselling session. At the end of the session, the STAI DY1 was repeated. If the subject opted for the predictive testing to proceed, a blood sample was obtained. As soon as the test result was available, a second session followed to discuss the result and its implications. In this second session, a repeat STAI DY1 was performed before and after counselling.

### Statistical methods

One researcher (ES) reviewed all semi-structured interviews, using open coding to identify recurring themes. This was done to obtain knowledge about the reactions of the participating subjects to the study procedure.

Demographic variables are presented as numbers and percentages for categorical data and as means with standard deviation for symmetrically distributed continuous variables. A family-clustered logistic and multinomial logit model was used to model the outcome variable that categorizes the participating status of the at-risk relatives into the dichotomous groups participation versus no-participation on the one hand and into a three level outcome variable on the other hand: participation into phase I, phase II or no participation. Both models investigate the effect of the degree of relationship of the relatives to the proband and gender of the at-risk relative on the participation status, taking family cluster into account. For the subset of participating subjects, the odds of participating in phase II compared to phase I are estimated using generalized linear models with binomial distribution and logit link function, clustered for family and controlling for subjects’ characteristics.

The safety of the new intake procedure is investigated through mixed model analysis of the mean STAI DY1 score, controlling for STAI DY2 at baseline and *BRCA* test result.

Statistical analyses were performed using IBM SPSS Statistics for Windows, Version 22.0 (Armonk, NY: IBM Corp) and the GLIMMIX procedure in SAS, version 9.3 (SAS Institute Inc., Cary, NC, USA).

## Results

### Characteristics of study population, uptake of counselling and predictive testing

Twenty families were included. The total number of relevant at-risk relatives eligible for the study was 172. Characteristics of the study population are shown in Table 1.

**Table 1:** Description of the study population

	Total study population	Participating subjects	Participating subjects phase I	Participating subjects phase II	Real decliners
Nr. of families	20 (19 *BRCA1/*1 *BRCA2*)				
Nr. of family members	172	89/172 52 %	47/89 53 %	42/89 47 %	59^a^/172 34 %
**Participating subjects phase II**					
*After letter*				28/42 67 %	
*After letter and phone call*				14/42 33 %	
Female	87/172 51 %	53/87 61 %	33/53 62 %	20/53 38 %	27/87 31 %
**Relation to proband**					
*First degree*	48/172 28 %	30/48 63 %	19/30 63 %	11/30 37 %	17/48 35 %
*Second degree*	31/172 18 %	21/31 68 %	12/21 57 %	9/21 43 %	3/31 10 %
*More than 2 degree*	93/172 54 %	38/93 41 %	16/38 42 %	22/38 58 %	39/93 42 %
**Subset of participating subjects**					
Age (year)		46 (17)	44 (17)	48 (16)	
Mean (SD) [min–max]		[18–77]	[18–77]	[18–75]	
Children		59/89 66 %	30/59 51 %	29/59 49 %	
Education level^b^		A = 13/89 (15 %)	A = 6/47 (13 %)	A = 7/42 (17 %)	
		B = 23/89 (26 %)	B = 16/47 (34 %)	B = 7/42 (17 %)	
		C = 40/89 (45 %) D = 13/89 (15 %)	C = 19/47 (40 %) D = 6/47 (13 %)	C = 21/42 (50 %) D = 7/42 (17 %)	

Description of characteristics of study population. Percentages given for ‘participating subjects’ and ‘real decliners’ with reference to the ‘total study population’. Percentages given for ‘participating subjects phase I & II’ with reference to the ‘participating subjects’

^a^24/172 (14 %) at-risk family members could not be reached

^b^Used codes for ‘education level’: A = primary education; B = secondary education; C = higher education; D = university level

Forty-seven (53 %; 95 % CI 43–63) relatives came forward for predictive counselling through phase I, which means that 27 % (47/172) of the total study population followed the standard procedure. Of these 47 relatives, 46 (98 %) decided to have a predictive genetic test. Eighteen relatives (39 %; 95 % CI 26–54) were carrier of a *BRCA* mutation.

Forty-two (47 %; 95 % CI 37–57) relatives came forward through phase II, which means that 24 % (42/172) of the total study population was reached by the intervention phase. So, 34 % (42/125, 95 % CI 26–42) of relatives were additionally reached, compared with the standard procedure. Of these 42 relatives, 41 (98 %) decided to have a predictive genetic test. Twelve family members (29 %; 95 % CI 18–44) were carrier of a *BRCA* mutation.

### Characteristics of non-participating relatives

Of the 172 at-risk relatives, 83 (48 %; 95 % CI 41–56) did not participate in the study. Thirty- four (41 %) of them were females. Eighteen (22 %) were first degree relatives towards the proband, 10 (12 %) second degree, and 55 (66 %) were more than second degree relatives.

The informative letter was sent only to 59 (71 %) of them (real decliners), because we did not succeed in getting coordinates of the remaining 24 (29 %). Sixteen (27 %) returned the reply form. None of these seemed to have been disturbed by the letter. Thirty-five of the remaining 43 could be contacted by phone, and therefore we were able to probe for the motivation in 51 (61 %) of the non-participants, for not participating. Most important reasons were: having already a preventive program (31 %), being childless (24 %), being not interested (18 %) and age (16 %).

### Family-averaged participation probabilities and characteristics

The family-averaged predicted probability for participation in the counselling procedure (phase I + II) is 56 % (95 % CI 41–70). This estimated probability increases to 62 % (95 % CI 50–72) if only contacted at-risk relatives were included. More specific this study focusses on the additional participation of at-risk relatives through phase 2, for which the family-averaged predicted probability for participation is 25 % (95 % CI 15–36), or 28 % (95 % CI 18–39) considering only contacted at-risk relatives.

A family-clustered analysis reveals that women participate more often in the counselling procedure (phase I + II) compared to men (*p* = 0.014, OR 2.48, 95 % CI 1.21–5.11). Participation depends also on ‘degree of relationship to the proband’ (*p* = 0.027). First and second degree relatives participate more than higher degree relatives (first degree: OR 3.18,95 % CI 1.22–8.25, *p* = 0.018; second degree: OR 3.76, 95 % CI 1.17–12.11, *p* = 0.027).

Family-averaged estimated participation probabilities are shown in supplementary table S1.

Second, a baseline category multinomial logit model was used to model the outcome variable that categorizes the participating status of all at-risk relatives into participation into phase I, phase II (reference category), or no participation (supplementary table S2). The odds of participating in phase I compared to phase II is significant higher for women compared to men (OR 6.32, 95 % CI 2.00–19.92, *p* = 0.002). Also, the odds of ‘participating’ in phase I compared to phase II is higher for first degree relatives than for more than second degree relatives (OR = 11.87, 95 % CI 2.48–56.78, *p* = 0.002).

### Results of the STAI

**Fig. 2:**
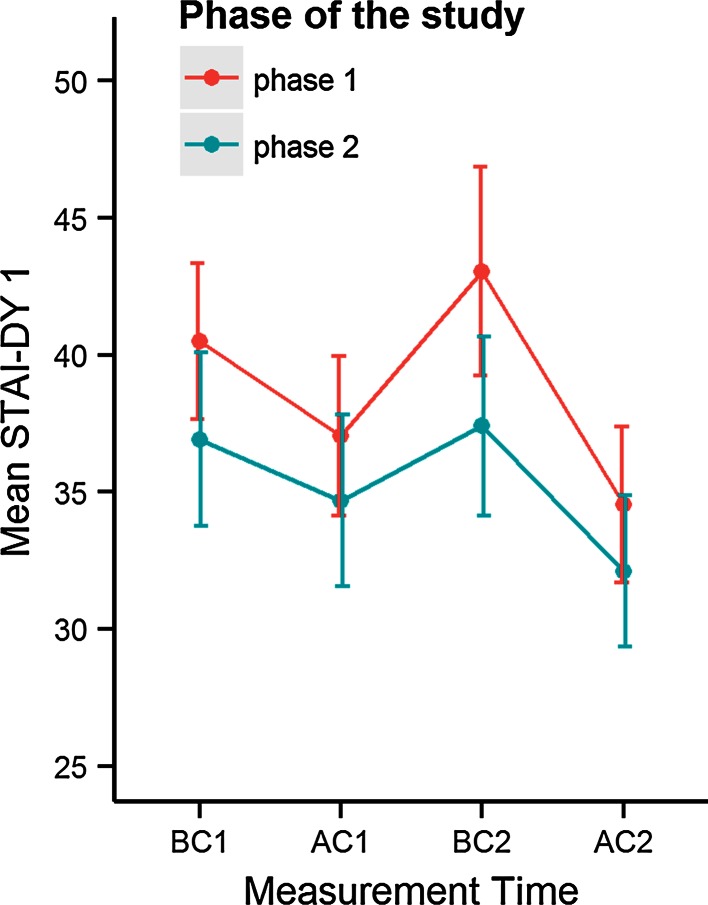
Fluctuation in the observed mean value of STAI DY1 score by phase. *Error bars* displayed are 95 % CI for the unadjusted mean STAI DY1 score. *BC* before consultation, *AC* after consultation

The estimated family-clustered mean STAI DY1 score before first consultation, for a participating relative with mean STAI DY2 score from 37.62, is 39.29 (SE 1.22) in the first phase, and 38.22 (SE 1.36) in the second phase (estimated mean difference in STAI DY1 value between phase I and II is 1.07; *p* = 0.533; 95 % CI −2.35 to 4.48). A fluctuation could be seen in the longitudinal analysis for the mean value of STAI DY1 score for all participating relatives before and after the first and second consultation (Fig. 2). This fluctuation of the STAI DY1 score before and after the second consultation is ‘test result-dependent’ (*p* = 0.004), controlling for study phase and STAI DY2 score of participating relatives. Before the first consultation the mean STAI DY1 score was statistically significant higher than after the first consultation (negative test result: estimated mean change before versus after consultation 3.42 STAI DY1 points, 95 % CI 0.77–6.08; positive test result: 3.64 STAI DY1 points, 95 % CI 0.46–6.82). If the participating subject is a carrier, there is no drop observed in the level of anxiety after discussion of the result (estimated mean change before versus after consultation 1.01 STAI DY1 points, 95 % CI −2.17 to 4.19), while a drop of 10.26 STAI DY1 points (95 % CI 7.44–13.10) is observed if the participating subject received a negative *BRCA* result (*p* < 0.001) (supplementary figure S1a).

Participating relatives with the same value of STAI DY2 could not be proven more or less anxious/stressed at the same moment of counselling in phase II in relation to phase I, controlling for test result (estimated mean change in STAI DY1 in phase II compared to phase I, −2.69 STAI DY1 points, *p* = 0.054, 95 % CI −5.42 to 0.04) (supplementary figure 1b/c).

## Discussion

This study addressed an important topic, namely directly informing relatives at risk for hereditary breast/ovarian cancer in known *BRCA* families about the familial cancer risk, the presence of a *BRCA* mutation and the availability of predictive genetic testing. The results of the study demonstrated the efficacy, feasibility and safety of this strategy. By literature search only a few other studies could be found, in which at-risk relatives for ‘adult onset’ familial cancer syndromes were also directly informed [13–15]. In the Australian study of Suthers et al. [15], letters were also directly sent to at risk relatives, but baseline (no intervention) measures were provided by a historical cohort, and only close relatives were taken into account. Evans et al. [14] studied a direct approach in first generation relatives of *BRCA* carriers, by sending letters firstly to the general practitioners (GP), and after the GP’s consent, to the relatives their selves. In the study of Aktan-Collan et al. [13], high risk family members of families with Lynch syndrome were also directly contacted by sending letters. This study started from the non-directive counselling model mainly using the proband to dissiminate information to the family, which is adopted by most genetic counsellors. Is was previously shown that the transfer of information from probands to their relevant relatives is highly defective [7]. The proband often perceives the disclosure process as difficult and stressful [8]. It is this kind of obstacles that prevents proper information dissemination. On the other hand, we also showed that a good understanding of the personal risk by at risk relatives, leads to a high uptake of predictive genetic testing [7], which was consistently observed in this study: nearly all of the participating subjects decided to have a predictive genetic test, of which 34 % were carrier of a *BRCA1/2* gene mutation.

Most of the literature concerning the process of genetic counselling has the premise that genetic counsellors are not allowed to ‘violate’ the proband’s privacy. There has been already an extensive debate about the apparent conflict between the respect for the privacy of the proband, and the rights of the relatives to be notified about important genetic information [16]. The actual availability of effective screening/prevention for *BRCA* mutation carriers (including prophylactic mastectomy, prophylactic oophorectomy, chemoprevention), of specific treatments, and of the possibility of PGD with reproductive implications, makes ‘the right to know’ a very major and dominant issue [17–23]. The debate that raised in the literature and the studies looking for a novel approach, demonstrate that there is an urgent need to develop more efficient, but acceptable procedures for informing at-risk relatives.

### Efficacy of the study

This study shows that adopting a stepwise interventional approach in which informative letters are sent to at-risk relatives, is *efficient* in increasing the rate of uptake of predictive genetic counselling and testing, compared to the ‘proband-only’ driven information. The percentage of at-risk relatives which came forward nearly doubled after intervention. Finally, 52 % of the at-risk relatives were counselled, of which nearly all (98 %) performed a predictive genetic test. Thirty relatives turned out to be carrier of a *BRCA1/2* mutation, and could therefore engage in effective prevention. The family-averaged predicted probability for participation in the counselling program (phase I and II) is 56 %. This study focusses on the additional participation of at-risk relatives through the interventional phase, for which the family-averaged predicted probability for participation is 25 %. These results proof the important gain in uptaking at-risk relatives in the procedure of predictive counselling/testing by using a novel interventional approach. These results are consistent with the study by Suthers et al. [15], in which the average proportion of relatives whose genetic status was clarified in the baseline cohort was 23 %, while in the intervention cohort, this proportion was 40 %. Women participate more often in the counselling program (phase I and II), compared to men (*p* = 0.014). This is concordant with other studies which demonstrated a generally higher uptake among women. In the study by Evans, uptake of predictive testing after a directive approach was as high as 74 % for females and 42 % for males in first generation, which decreased to 44 % of women and 9 % of males in the second generation [14]. Male relatives have been shown already to be less frequently informed about HBOC than female relatives, and are less involved in the family communication process [14, 24]. The odds of ‘participating’ by a conventional procedure is also statistically significant higher for women compared to men. Participation also depends on ‘degree of relationship to the proband’ (*p* = 0.027). First and second degree relatives tend to participate more than higher degree relatives. The odds of participating by a standard procedure is higher for first degree relatives than for second degree relatives (*p* = 0.002). Using a novel interventional counselling approach leads to a higher uptake of predictive genetic counselling/and testing.

### Feasibility of the study

The cooperation of the proband remains important, to ensure the feasability. The proband describes initially the structure of the family pedigree, and also provides the necessary information. Without this cooperation, there are still impassable limitations. Most of the probands did cooperate well, and experienced the intervention as positive, as it takes over part of their difficult function as being ‘messenger of complex information’ in the family, which is often experienced as an additional stressful burden, especially when it comes to more distant family. In families with a general poor communication style, and in which the proband had rather superficial emotional ties with other relatives, a process of ‘cascade’ counselling was observed. If one other relative was reached, this relative provided then further additional information, giving us the possibility to get through to other family members. For some of the relatives, sending a letter was not efficient enough. Eventually, 33 % of the participating relatives in the interventional phase, came forward only after a final phone call. *So, having an even more direct contact, leads to a higher uptake of predictive counselling and testing.* A remaining obstacle in the study is the percentage of relatives which could not be reached, because of lack of coordinates (14 %). The approach of directly contacting at risk relatives is obviously more easy in countries which have registries that can facilitate active recruitment, such as Finland and Denmark [13].

### Safety/acceptability of the study

Nearly all the participating subjects which came forward by the intervention phase, experienced the directly sent letter/phone call as being positive, and important. They found the letter a real trigger to make an appointment, and preferred directly provided correct information by a MD. They also mentioned that the letter also was the origin of a cascade of communication in the family. Some of the participating relatives stated that it would be a criminal negligence not to be informed. Researchers have not remarked visible adverse reactions towards the direct approach, and high levels of satisfaction were reported. The possibility of preventing a serious disease was experienced as positive.

For participating relatives that have the same value of STAI DY2, no significant differences in the mean STAI DY1 score measured before the first consultation could be found between phase I and II. This demonstrates that the intervention did not cause any more anxiety or stress. Participating relatives with the same value of STAI DY2 could also not be proven more or less anxious/stressed at the same moment of counselling in phase II in relation to phase I, controlling for test result. These results demonstrate that the study procedure is psychologically safe.

One-third of the at-risk relatives in this study declined to participate for various reasons, which can be considered as a limitation of the study. The used interventional procedure should be improved, to diminish this part of decliners. The informative letter could be simplified, to be convinced that all receivers can well understand the content of the letter. A link could be created to a webpage of the Familial Cancer Clinic providing more detailed information, and a phone call could be made in a standard way a few weeks after sending the informative letter.

## Conclusion

The results of this study support an adaptation of the international guidelines on counselling strategies in families with a *BRCA* mutation, in which informing other family members at-risk is not only discussed or put forward as a potential option. The efficacy and safety of such intervention as demonstrated in this study and its utility should make such effort part of mandatory guidelines. It does not seem acceptable that such important information is withheld from high risk individuals at a time where effective prevention is available and also has potential therapeutic implications. These conclusions should not be restricted to HBOC but could also be extended to predictive genetic testing for other hereditary cancers or other important diseases that are preventable and for which effective treatments exist.

This study shows that there are persuasive arguments for a more family-oriented view of genetic information in which this information is also property of the relatives, and should and can be shared safely with other at-risk relatives. In the current study, it was our experience that it is perfectly possible to safeguard the intra-familial privacy of each individual relative. We would suggest to work in two different steps: first, the proband should be supported with written information to inform other relatives and second, an informative letter can be sent safely to the other at risk relatives with a subsequent phone call. In the near future it should be investigated how to improve this step by step approach, to overcome the limitations that were encountered. Introducing this novel approach should lead to more uptake of predictive genetic testing, better prevention strategies and eventually to fewer fatal cancer cases within these families.

## Electronic supplementary material

Supplementary material 1 (DOCX 12 kb)

Supplementary material 2 (DOCX 12 kb)

Supplementary material 3 Fig. S1 Fluctuation in the observed mean value of STAI DY1 score. Error bars displayed are 95% CI for the unadjusted mean STAI DY1 score. BC: before consultation, AC: after consultation. Fig. 1a: Observed fluctuation of the mean STAI DY1 score by test result (TIFF 4153 kb)

Supplementary material 4 Fig. 1b: Observed fluctuation of the mean STAI DY1 score by phase and positive test result (TIFF 4153 kb)

Supplementary material 5 Fig. 1c: Observed fluctuation of the mean STAI DY1 score by phase and negative test result (TIFF 4153 kb)
