# Supplementary material for: The impact of an interventional counselling procedure in families with a BRCA1/2 gene mutation: efficacy and safety
Source: Fam Cancer. 2016 Jan 9;15:155–62. doi: 10.1007/s10689-015-9854-4 (PMC4803813; doi:10.1007/s10689-015-9854-4)
Supplement: Supplementary file 1 — Supplementary material 1 (DOCX 12 kb) [file 10689_2015_9854_MOESM1_ESM.docx]

**Table S2:** Family-averaged estimated participation probabilities

| **Gender** | **Degree of relationship**  **to proband** | **Family-averaged estimated participation probability** | **Lower**  **95% Confidence**  **Limit** | **Upper**  **95% Confidence**  **Limit** |
| --- | --- | --- | --- | --- |
|  |  |  |  |  |
| **Male** |  |  |  |  |
|  | first degree | 0.57 | 0.35 | 0.77 |
|  | second degree | 0.61 | 0.34 | 0.83 |
|  | > second degree | 0.30 | 0.15 | 0.51 |
| **Female** |  |  |  |  |
|  | first degree | 0.77 | 0.57 | 0.90 |
|  | second degree | 0.80 | 0.57 | 0.92 |
|  | > second degree | 0.51 | 0.31 | 0.71 |
