# Supplementary material for: The impact of an interventional counselling procedure in families with a BRCA1/2 gene mutation: efficacy and safety
Source: Fam Cancer. 2016 Jan 9;15:155–62. doi: 10.1007/s10689-015-9854-4 (PMC4803813; doi:10.1007/s10689-015-9854-4)
Supplement: Supplementary file 2 — Supplementary material 2 (DOCX 12 kb) [file 10689_2015_9854_MOESM2_ESM.docx]

**Table S3:** Estimated odds ratios based on a baseline category multinomial logit model used to model the outcome variable that categorizes the participating status of all at-risk relatives into participation into phase I, phase II (reference category) or no participation

| **Outcome** | **Covariate** | **Odds ratio estimates** | **Lower 95% Confidence**  **Limit** | **Upper 95% Confidence**  **Limit** | **p-value** |
| --- | --- | --- | --- | --- | --- |
| Not participating vs. phase II | first vs. > second degree | 0.68 | 0,22 | 2,09 | 0.49 |
| Phase I vs. phase II | first vs. > second degree | 11.87 | 2.48 | 56.78 | 0.002 |
| Not participating vs. phase II | second vs. > second degree | 0.34 | 0.09 | 1.26 | 0.11 |
| Phase I vs. phase II | second vs. > second degree | 2.64 | 0.48 | 14.43 | 0.26 |
| Not participating vs. phase II | female vs. male | 0.72 | 0.31 | 1.69 | 0.45 |
| Phase I vs. phase II | female vs. male | 6.32 | 2.00 | 19.96 | 0.002 |
