# Supplementary figures and images for: The impact of an interventional counselling procedure in families with a BRCA1/2 gene mutation: efficacy and safety
Source: Fam Cancer. 2016 Jan 9;15:155–62. doi: 10.1007/s10689-015-9854-4 (PMC4803813; doi:10.1007/s10689-015-9854-4)

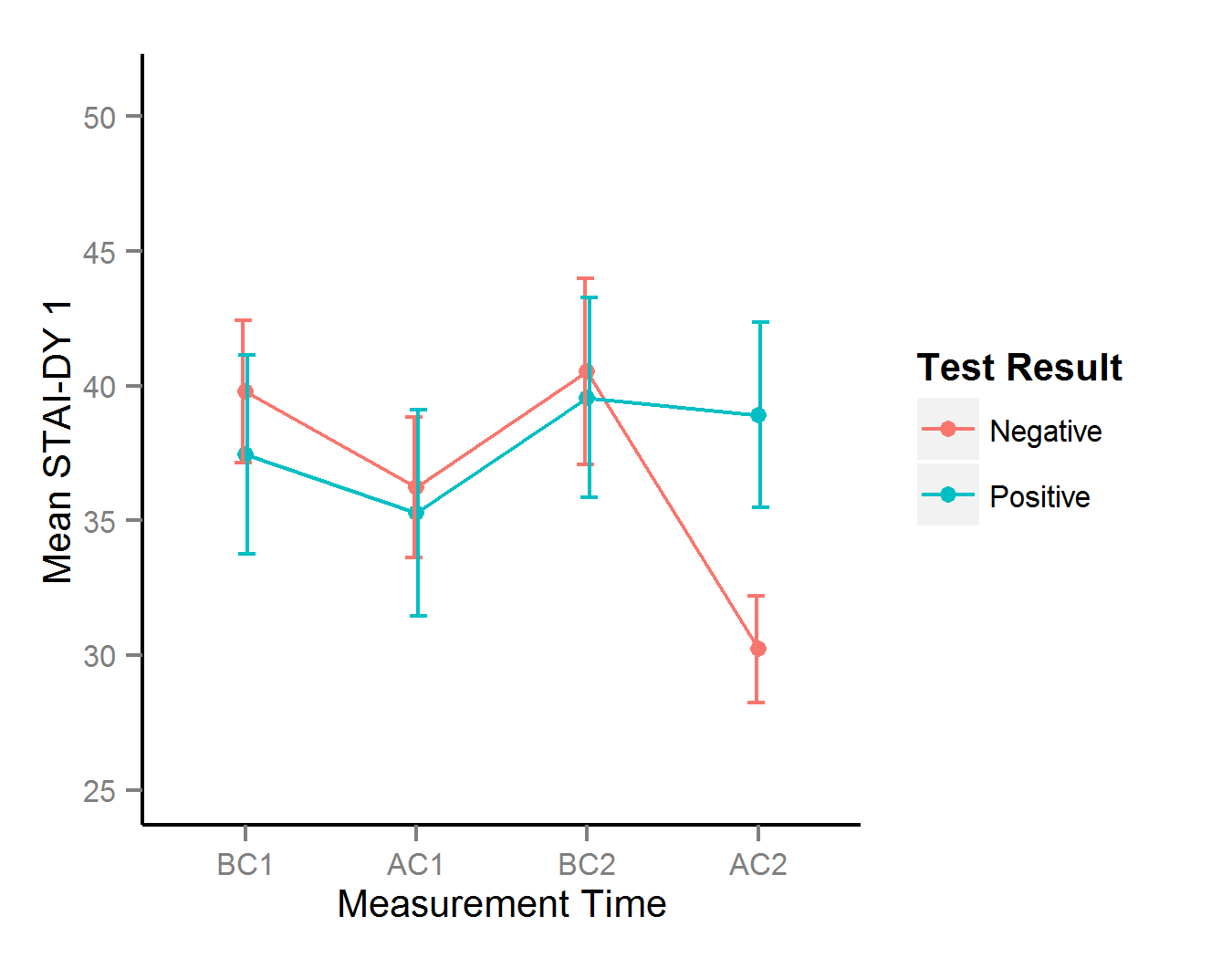

Supplement: Supplementary file 3 — Supplementary material 3 Fig. S1 Fluctuation in the observed mean value of STAI DY1 score. Error bars displayed are 95% CI for the unadjusted mean STAI DY1 score. BC: before consultation, AC: after consultation. Fig. 1a: Observed fluctuation of the mean STAI DY1 score by test result (TIFF 4153 kb) [file 10689_2015_9854_MOESM3_ESM.tiff]

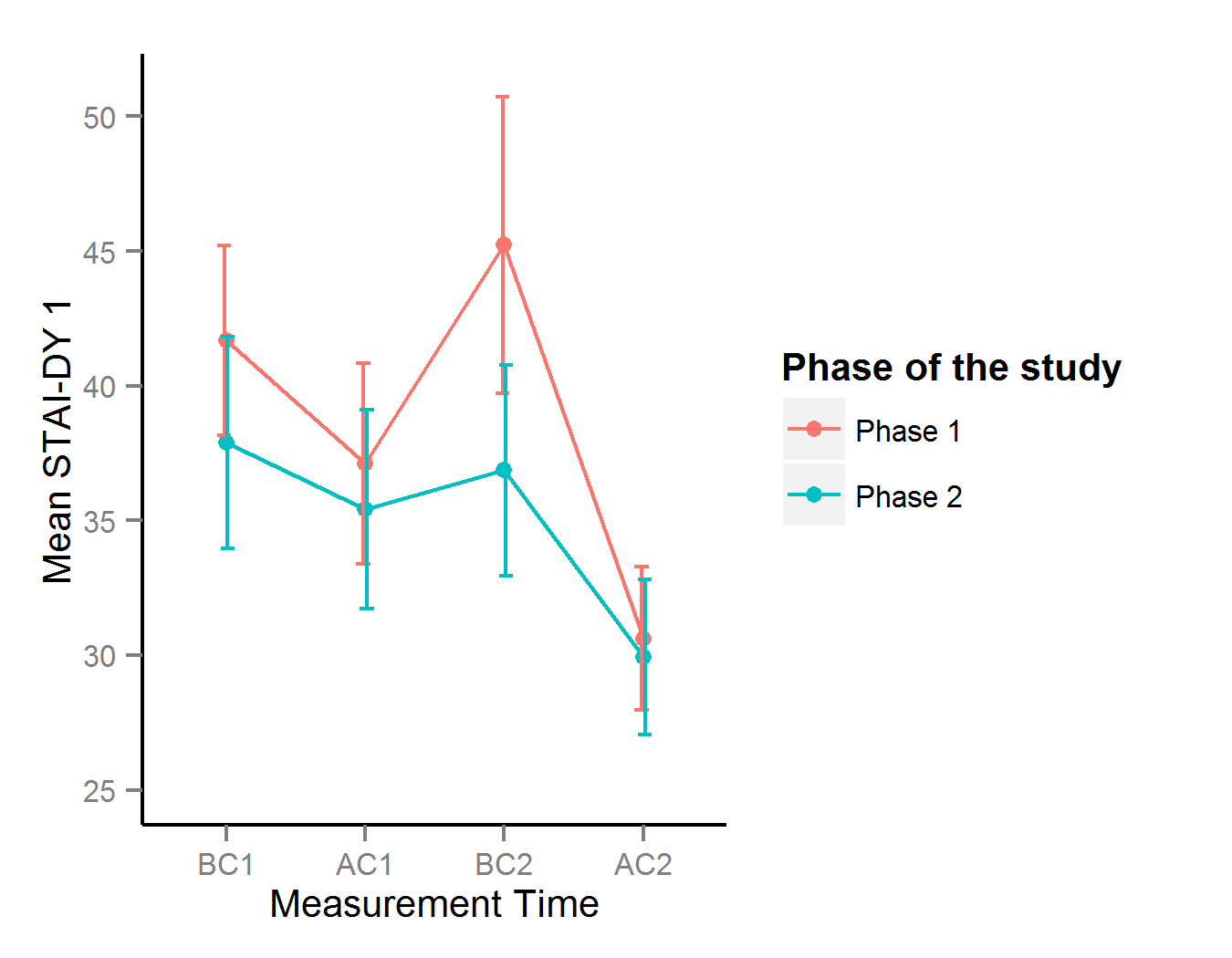

Supplement: Supplementary file 4 — Supplementary material 4 Fig. 1b: Observed fluctuation of the mean STAI DY1 score by phase and positive test result (TIFF 4153 kb) [file 10689_2015_9854_MOESM4_ESM.tiff]

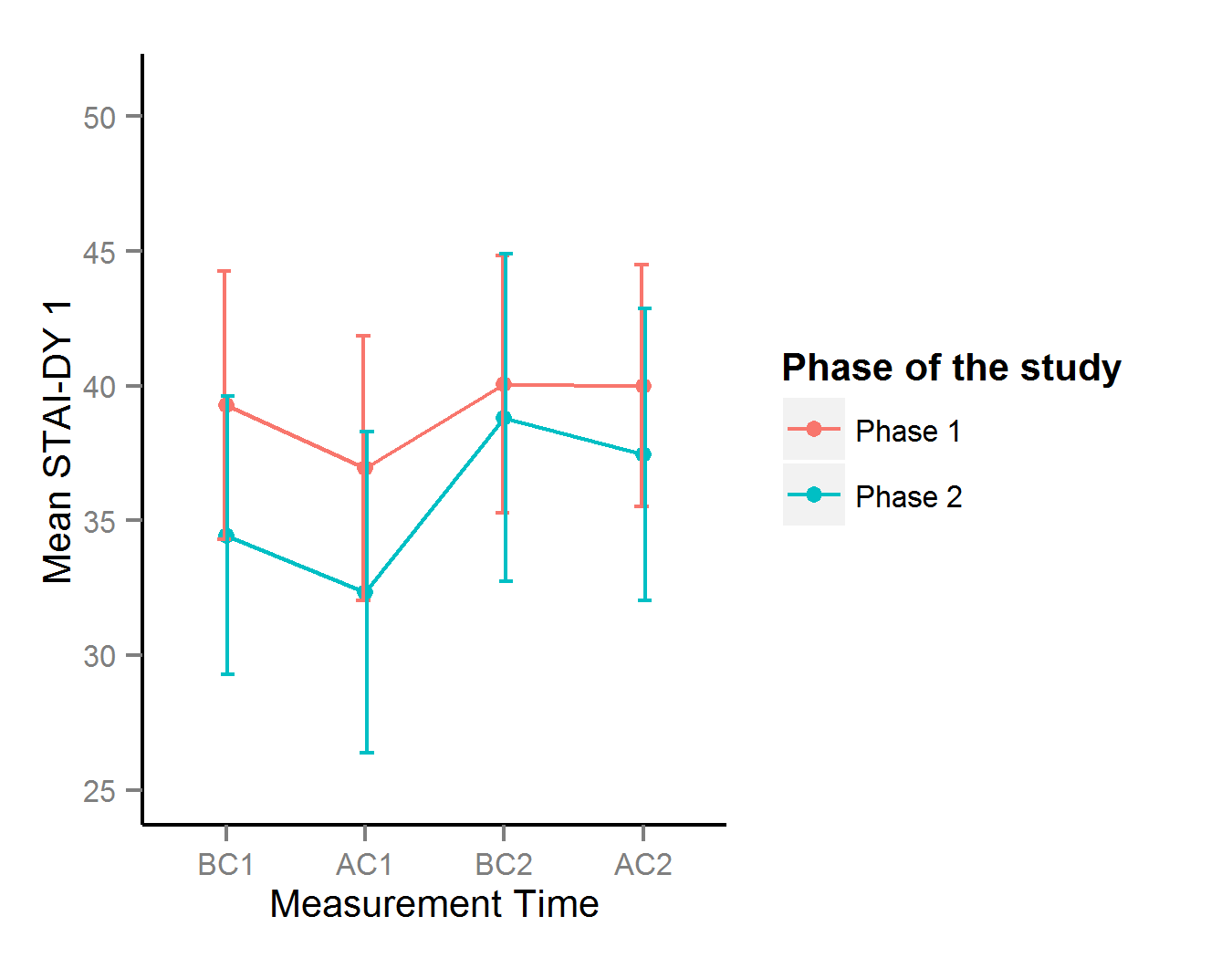

Supplement: Supplementary file 5 — Supplementary material 5 Fig. 1c: Observed fluctuation of the mean STAI DY1 score by phase and negative test result (TIFF 4153 kb) [file 10689_2015_9854_MOESM5_ESM.tiff]
